# A Nonparametric Bootstrap Confidence Interval for the Prevalence of Undiagnosed Type 2 Diabetes Mellitus in Hamadan, West of Iran

**Published:** 2018-08-11

**Authors:** Elham Azizi, Shiva Borzouei, Ali Reza Soltanian

**Affiliations:** ^1^ Department of Biostatistics, School of Public Health, Hamadan University of Medical Sciences, Hamadan, Iran; ^2^ Department of Internal Medicine, School of Medicine, Hamadan University of Medical Sciences, Hamadan, Iran; ^3^ Modeling of Noncommunicable Diseases Research Center, School of Public Health, Hamadan University of Medical Sciences, Hamadan, Iran

**Keywords:** Non-parametric bootstrap, Statistical model, Diabetes mellitus, Epidemiology, Iran

## Abstract

**Background:** The prevalence of type 2 diabetes mellitus (T2DM) is increasing in Iran. This study determined the prevalence of undiagnosed type 2 diabetes mellitus in apparently normal individuals in Hamadan, west of Iran.

**Study design:** A cross-sectional study.

**Methods:** A sample of 106 apparently normal volunteers aged 18 yr and more were enrolled, and T2DM was diagnosed using hemoglobin A1c (HbA1c) levels from 2015-2016. The nonparametric bootstrap method was used to eliminate the undesirable effect of small sample size on the estimation of standard error of multiple logistic regression coefficients and confidence interval for the prevalence of undiagnosed T2DM.

**Results:** Overall, 23 (21.69%) were male. The mean (±sd) age of the participants was 43.76 ±14.01 year. In 78.3% of individuals, HbA1c level was within normal range (<5.7), 13.21% was in the range of 5.7-6.4 (undiagnosed pre-T2DM), and 8.49% was ≥6.5 (undiagnosed T2DM). Multiple logistic regression gave the characteristic distribution of volunteers such as sedentary hour (P=0.001), family history of diabetes (P=0.001), smoking (P=0.002), and age (P=0.012) had the odds on the significant effect on undiagnosed T2DM.

**Conclusions:** The prevalence of undiagnosed T2DM among apparently normal individuals in Hamadan was relatively high. Addition to age, factors such as sedentary, exposure to smoking and having a history of diabetes in family can be a prognosis for undiagnosed T2DM in apparently normal individuals

## Introduction

Type 2 diabetes mellitus (T2DM) is a chronic metabolic and non-contagious disease spreading worldwide ^1^. Type 2 diabetes plays a role in losing years of human’s life, and it was the cause 1.5 million deaths worldwide in 2013^2^. The prevalence of undiagnosed type 2 diabetes mellitus (UT2DM) is different in countries. The prevalence rate of UT2DM in Colombia among adult (i.e., age ≥35 yr) was 2.59% ^3^, in Jordan 46.4% ^4^, and in two Spanish surveys^5^, 1.95% (ENINBSC survey) and 2.88% (CRONICAS cohort study).

The frequency of people with diabetes in 2014 was approximately 422 million projected to increase to 438 million by 2030^6^. Moreover, centers for disease control and prevention (CDC) reported that the number of diagnosed and undiagnosed diabetes in the United States in 2017 was 23.1 million and 7.2 million, respectively ^7^. The prevalence of diagnosed T2DM in Yazd Province was estimated at 16.3% in 2012 ^8^.

Iran had the third rank among the countries of the Middle East and North Africa in terms of the prevalence of diabetes mellitus after Saudi Arabia and Egypt in 2011 ^9^. Iran is expected to maintain its status for the prevalence of diabetes mellitus in 2030 ^9^. Although Iran was not among the top 10 countries with T2DM until 2030 in the world, according to IDF forecast the prevalence of T2DM will be twice (~8.3 million) in Iran until 2030 ^9^. Healthcare expenditure for T2DM is high and this cost was estimated at USD$ 548 billion in 2013 ^9^. A family in low- and middle- income countries is not able to afford such expenditures. 83.8% of people with UT2DM live in low- and middle- income countries ^10^.

Now, the various aspects of diabetes mellitus such as prevalence, people susceptible to T2DM, symptoms of T2DM, and ways to control and its prevention should be addressed. If this disease is not controlled, all countries especially low- and middle- income countries will be in crisis.

We aimed was to estimate the prevalence of UT2DM in apparently normal individuals in Hamadan, west of Iran. Additionally, the nonparametric bootstrap method was used to eliminate the undesirable effect of small sample size on the estimation of standard error of multiple logistic regression coefficients and confidence interval for the prevalence of undiagnosed T2DM.

## Methods

### Dataset

Dring the Hamadan diabetes risk assessment study, whose goal was to create an appropriate tool for diagnosis of individuals at risk for T2DM (age ≥18 yr), 130 normal volunteers were invited by the simple sampling method. They were referred to the Hamadan Diabetes Center as a patient’s companion. Of the volunteers (n=130), only 106 had their hemoglobin A1c (HbA1c) measured at the laboratory from Nov 27, 2015, to Mar 15, 2016.

The inclusion criteria for apparently normal subjects were age 18 yr and more; no mental disability; no history of type 1 diabetes, T2DM, or gestational diabetes; no current pregnancy (for women); and no current use metformin or other glucose control drugs.

Individuals were referred to the laboratory for HbA1c test, and the diagnosis of individuals as having or not having diabetes was made based on the HbA1c results from an endocrinologist.

We applied the American Diabetes Association criteria to the hemoglobin A1c results with cut-off points of less than 5.7% (<40 mmol/ mol) as normal, 5.7%-6.4% (40-46 mmol/ mol) as pre-diabetes, and 6.5% and more (48 mmol/ mol) as indicative of type 2 diabetes ^11^.

Informed consent was obtained from all volunteers included in the study, and the Ethical Committee of Hamadan University of Medical Sciences approved the study (IR.UMSHA.REC.1394.238).

Addition to HbA1c, other individual’s characteristics such as age (year), sex (male or female), smoking status (never, former or current), height (m), weight (kg), waist circumference (cm), Sedentary (<5 or ≥ 5 h), family history of diabetes (yes or no), walking (< 30 or ≥30 min/day), and body mass index (BMI, <25 or ≥25 kg/m^2^) were measured.

### Statistical analysis and bootstrapping

The mean± standard deviation used to describe the quantitative variables, and frequency (percent) to describe the qualitative variables. The non-parametric bootstrap method applied to determine standard error and confidence interval of multiple logistic regression parameters and statistical significance levels. The pros and cons of bootstrap confidence intervals were mentioned in previous studies^12,13^. In this study, 106 re-samples (i.e., n=106) were selected by replacement random sampling, at 1000 times (i.e., B=1000). Multiple logistic regression coefficients, standard errors, and significant levels were estimated based on generating bootstrap samples as following procedure.

Using a scenario proposed^14^, an algorithm for non-parametric confidence interval can be written as follows:

First, make a dataset for dichotomous response with covariates (e.g., sex=x_1_, BMI=x_2_, age=x_3_, waist circumference=x_4_, sedentary=x_5_, family history of diabetes=x_6_, smoke=x_7_, and walking=x_8_). Therefore, multiple logistic regression will be,$\log it[\pi_{i}]=\beta_{0}+\sum_{i=1}^{8}\left(\beta_{i}x_{ij}\right)$, where, j denotes individuals (j=1,2,…,n).

Second, draw a bootstrap sample by sampling the pairs (i.e., dependent and covariates) with replacements sampling method from above dataset, i.e.,

(y, x_1_, x_2_,x_3_,x_4_,x_5_,x_6_,x_7_,x_8_)_b_*=

[(y_11_, x_11_, x_21_,x_31_,x_41_,x_51_,x_61_,x_71_,x_81_ ,…, y_1n_, x_1n_, x_2n_,x_3n_,x_4n_,x_5n_,x_6n_,x_7n_,x_8n_)]_b_

where, b=1,2,…,B denotes the number of bootstrap sample, and x_ij_ denotes a value for i^th^ covariate and j^th^ individual.

Third, for each b=1,2,…,B estimate the bootstrap sample statistics${\hat{\theta }}_{1}*,\;{\hat{\theta }}_{2}*,...,{\hat{\theta }}_{1}*\;{\hat{\theta }}_{B}*,where,{\hat{\theta }}_{b}*=({\hat{\beta }}_{0}*,{\hat{\beta }}_{1}*,...,{\hat{\beta }}_{8}*,e^{{\hat{\beta }}_{0}*},e^{{\hat{\beta }}_{1}*},...,e^{{\hat{\beta }}_{8}*})$by refitting above logistic regression.

Fourth, estimate the bootstrap mean and standard error of

$${\hat{\theta }}_{b}*=({\hat{\beta }}_{0}*,{\hat{\beta }}_{1}*,...,{\hat{\beta }}_{8}*,e^{{\hat{\beta }}_{0}*},e^{{\hat{\beta }}_{1}*},...,e^{{\hat{\beta }}_{8}*})$$

as follows,

$$E(\theta *)=\frac{1}{B}\sum_{b=1}^{B}{\hat{\theta }}_{b}*and\;se(\theta *)=\sqrt{\frac{1}{B}\sum_{b=1}^{B}\left({\hat{\theta }}_{b}*-E(\theta *)\right)}.$$

Fifth, estimate (1-α)100% bootstrap confidence interval by finding quintile of bootstrap replicates.

Sixth, determine bootstrap *P*-value by $p=\frac{1+\left(\neq ()\left|{\hat{\theta }}_{b}*\right|>\left|\hat{\theta }\right|\right)}{B+1}\;where\;{\hat{\theta }}_{b}^{*}=({\hat{\beta }}_{b}^{*},t_{\beta_{1}}^{*})$ denotes parameter estimation under the fourth step and$\hat{\theta }=({\hat{\beta }}_{b}^{},t_{\beta b}^{})$ denotes the parameter estimation of the logistic model using the observed data for b=1,2,…,B. The statistical analysis was performed using R 3.2.2. The statistical significance level was less than 0.05.

## Results

The data consisted of 106 apparently normal individuals. The aged range of the subjects was 23-73 yr old with a mean (±sd) 43.76±14.01 yr. Approximately, one-third of subjects were over 40 yr old. The frequency of male and female was 23 (21.69%) and 83 (78.30%), respectively. 36.79% (n=39) had overweight (i.e., 25≤BMI≤29 kg/m^2^) and 7.55% (n=8) had an obesity (i.e., BMI≥30 kg/m^2^). Waist circumference of 55.66% (n=59) was higher than the standard level (i.e., Male≥90 and female≥80 cm).

Table 1 shows the distribution of participants under demographic characteristic levels. All participants were categorized into the three groups normal (78.30%; 95%CI_boot_: 71.56%-85.04%), undiagnosed pre-T2DM, (13.21%; 95%CI_boot_: 12.34%-14.08%) and undiagnosed T2DM (8.49%; 95%CI_boot_: 7.43%-9.55%) based on HbA1c levels. The total prevalence of undiagnosed pre-T2DM and undiagnosed T2DM together was 21.7% (95%CI_boot_: 19.88%-23.52%)

**Table 1:** Distribution of normal, pre-diabetes and diabetes individuals for demographic character levels

**Variables**	**Normal (n=83)**		**Pre-diabetes (n=14)**		**Diabetes (n=9)**	
	**Number**	**Percent**	**Number**	**Percent**	**Number**	**Percent**
Sex						
Male	13	15.7	6	42.9	4	44.4
Female	70	84.3	8	57.1	5	55.6
BMI (kg/m^2^)						
<25	53	63.9	3	21.4	3	33.3
25-29	24	28.9	9	64.3	6	66.7
≥30	6	7.2	2	14.3	-	-
Age (yr)						
20-39	64	77.1	3	21.4	2	22.2
40-59	13	15.7	7	50.0	5	55.6
≥60	6	7.2	4	28.6	2	22.2
Waist circumference (cm)						
Male<90, female<80	44	53.0	2	14.3	1	11.1
Male≥90, female≥80	39	47.0	12	85.7	8	88.9
Sedentary time ^a^ (hours)						
<5	50	60.2	8	57.1	5	55.6
≥5	33	39.8	6	42.9	3	44.4
Family history of diabetes						
Yes	10	12.1	9	64.3	7	77.8
No	73	87.9	5	35.7	2	22.2
Smoke status:						
Never	66	79.5	6	42.9	3	33.3
Former	8	9.6	3	21.4	2	22.2
Current	9	10.8	5	35.7	4	44.4
Walking (min/day)						
<30	35	42.2	8	57.1	2	22.2
≥30	48	57.8	6	42.9	7	77.8

^a^ Sedentary denotes tending to spend much time seated or lying down (i.e., somewhat inactive) while working at home or office, watching television, and other things.

In Table 2, without losing information we merged undiagnosed pre-T2DM (UPT2DM) and undiagnosed T2DM (UT2DM) subjects in a new group, then their demographic characteristics were compared with normal group using a nonparametric bootstrap multiple logistic regression model. The proportion of UT2DM (UPT2DM + UT2DM) in females was not significantly higher than in males (ORboot=1.14, 95%CI_boot_: 0.45-4.49). In addition, the proportion of UPT2DM+UT2DM in older people, i.e., over 40 yr old, was more than youngers, i.e., less than 39 yr old (OR_boot_=4.35, Table 2). Of the youngers (n=69), 3 (4.35%) had undiagnosed pre-diabetes, 2 (2.9%) had undiagnosed T2DM, and totally 5 (7.25%) had pre-UT2DM or UT2DM.

**Table 2:** Comparison of demographic characteristics between normal and undiagnosed diabetes using multiple logistic regression

**Variables**	**Standard estimation**		**Bootstrap estimation**	
	**Adjusted OR (95% CI)**	***P*** ** value**	**Adjusted OR 95% CI**	***P*** ** value**
Sex				
Male	1.00		1.00	
Female	1.19 (0.24, 5.81)	0.834	1.14 (0.45, 4.49)	0.812
BMI (kg/m^2^)				
<25	1.00		1.00	
≥25	2.02 (0.32, 12.86)	0.457	1.73 (0.47, 9.51)	0.433
Age (yr)				
<40	1.00		1.00	
≥40	6.59 (1.28, 16.04)	0.024	4.35 (1.91, 12.65)	0.012
Waist circumference (cm)				
Male<90, female<80	1.00		1.00	
Male≥90, female≥80	1.96 (0.22, 17.36)	0.54	1.95 (0.38, 10.22)	0.435
Sedentary time (hours)^a^				
<5	1.00		1.00	
≥5	8.62 (4.28, 17.33)	0.001	8.62 (1.54, 3.01)	0.001
Family history of diabetes				
Yes	1.00		1.00	
No	0.11 (0.05, 0.25)	0.001	0.11 (1.37, 3.43)	0.001
Smoke status				
Never	1.00		1.00	
Former + Current	9.98 (2.27, 24.80)	0.002	4.75 (1.95, 12.14)	0.003
Walking (min/day)				
<30	1.00		1.00	
≥30	0.41 (0.09, 1.76)	0.230	0.50 (0.11, 1.82)	0.281

^a^ Sedentary time was defined in terms of the amount of time (hours) a person sitting at the office or at home.

The rate of smoking among normal subjects was more than undiagnosed T2DM (*P*=0.002, Table 2). In addition, sedentary hour (*P*=0.001), family history of diabetes (*P*=0.001), and the individual’s age (*P*=0.012) were important prognosis for T2DM among apparently healthy subjects (Table 2).

## Discussion

The present study was conducted to estimate UT2DM in Hamadan, west of Iran for the first time. We also identified the demographic characteristics of subjects not diagnosed with type 2 diabetes. Moreover, HbA1c test was repeated to ensure that subjects are normal or diabetic. We obtained standard deviations using the nonparametric bootstrap method to control error due to small sample size.

We observed over the past 5 yr in Iran, no studies such as our study had been conducted on subjects having or not having UT2DM‏. In other words, there is no statistics or information on the prevalence or incidence of UT2DM in Iran in recent years. For this reason, we conducted this research.

We obtained the parameters of multiple logistic regression model and confidence intervals from the nonparametric bootstrap method in order to eliminate the problem of small sample size, so we improved the results, e.g., odds ratios and confidence intervals, using the nonparametric bootstrap method.

This study showed that the proportion of UT2DM in subjects aged ≥18 yr was 21.7% (i.e., 13.21% had undiagnosed pre-T2DM and 8.49% had UT2DM), while the proportion of UDM in Tehran was reported about 5.1% in subjects aged ≥20 yr in 2009 ^15^. The UT2DM in the west of Iran was more prevalent than the central region of Iran. This difference is due to the difference in the time of study in these studies.

The prevalence of undiagnosed pre-diabetes and UT2DM among 14815 individuals aged 18-69 in Bangladesh was 6.9% and 17.5%, respectively ^16^, while the prevalence of UT2DM in our study was approximately 3% less than Bangladesh ( 21.7% vs. 24.4%, respectively). Moreover, in Spain, some studies have been conducted to determine the prevalence of UT2DM, in which the prevalence was lower than in our study. In other words, in Madrid, Spain ^17^, the prevalence of UT2DM in people aged 45-74 yr was 7.4% and in the second Spanish study, it was 6% ^18^. Although the subjects in Spanish studies were older than those participated in our study, the prevalence of UT2DM in Spain was much lower than in our study. The proportion of UT2DM was approximately zero in Swedish children, but the population studied were children aged 11 to 13 yr ^19^.

We found that the prevalence of UT2DM in European countries was less than Asian countries (e.g., Iran and Bangladesh). Such a conclusion is obtained from our literature review, and for a more accurate conclusion, we need a systematic review and meta-study.

We determined the prevalence of UT2DM based on HbA1c, while some European studies have identified UT2DM based on oral glucose tolerance test (OGTT). The prevalence of HbA1c based UT2DM may be estimated less than OGTT^17^. Therefore, finding a higher prevalence of UT2DM in this study compares to some European studies^17-19^ may be due to a difference in laboratory testing, e.g., HbA1C and OGTT.

Bernabe-Ortiz et al^10^ showed that the prevalence of UT2DM was 52.8% by the HbA1c test, which was approximately 2.5 times more than the prevalence of UT2DM in our study. The prevalence of UT2DM in that study, was the results of four studies, three of which, were conducted in Asian countries (two studies in China, one study in India) and a study in South America (Mexico).

Despite our study, the prevalence of UDM in another study, in the elderly was lower than that of young people^10^. These differences moght be due to a different policy of governments in discovering patients and people at high risk.

In high-income countries, preventive policies are more important than low- and middle- income countries. Therefore, the lack of attention to aging problems and preventive health measures is one of the factors of the inability and inefficiency in the discovery of undiagnosed diabetes in developing countries.

Population growth in Hamadan is decreasing ^20^ and in the next few decades, Hamadan will face a problem of aging. In addition, the level of awareness on diabetes is relatively low in Hamadan and some parts of Iran ^21^. Therefore, if policymakers do not start a preventive action, such as screening for the discovery of UT2DM, the disease will be prevalent in these societies and the complications caused will bring a lot of financial burden to the family. According to the results of this study, we suggest family counseling services become more active in this regard.

In this study, the dataset of the Hamadan diabetes risk assessment study (HDRA) was used and it may seem small sample size. Because apparently healthy people come from different areas of Hamadan to the diabetes center, the generalized undiagnosed diabetes prevalence can be extended to the general population. However, the prevalence of undiagnosed diabetes is not exhaustive in terms of sex and other demographic variables. In this study, 24 volunteers (21 males and 3 females) did not go to the lab for glucose testing. This distorted the sex distribution.

## Conclusion

The prevalence of UT2DM among apparently normal individuals in Hamadan was relatively high. In addition to individual's age, factors such as sedentary, exposure to smoking and having a history of diabetes in their family can be a prognosis for undiagnosed diabetes in apparently normal individuals. In accordance with the study's results, a diabetes screening program should be planned in Iran and people with high risk of type-2 diabetes must be identified.

## Acknowledgements

The authors would like to thank the volunteers who participated in this study.

## Conflict of interest statement

No conflict of interest is declared

## Funding

The study was funded by the Vice-chancellor for Research and Technology, Hamadan University of Medical Sciences (No. 9511267143).

## Highlights

- The prevalence of undiagnosed type 2 diabetes in Hamadan was 21.7%.
- Undiagnosed type 2 diabetes is found among young people <40 year.
- A sedentary hour was also found as a meaningful prognosis on type 2 diabetes.
